# Immunolocalization of K_ATP _channel subunits in mouse and rat cardiac myocytes and the coronary vasculature

**DOI:** 10.1186/1472-6793-5-1

**Published:** 2005-01-12

**Authors:** Alison Morrissey, Erika Rosner, Jennifer Lanning, Lavanya Parachuru, Piyali Dhar Chowdhury, Sandra Han, Gwendolyn Lopez, XiaoYong Tong, Hidetada Yoshida, Tomoe Y Nakamura, Michael Artman, Jonathan P Giblin, Andrew Tinker, William A Coetzee

**Affiliations:** 1 Pediatric Cardiology, NYU School of Medicine, New York, USA; 2 Pharmacology, NYU School of Medicine, New York, USA; 3 Physiology and Neurosciences, NYU School of Medicine, New York, USA; 4 Department of Molecular Physiology, National Cardiovascular Center Research Institute, Osaka, Japan; 5 BHF Laboratories and Department of Medicine, University College London, UK

## Abstract

**Background:**

Electrophysiological data suggest that cardiac K_ATP _channels consist of Kir6.2 and SUR2A subunits, but the distribution of these (and other K_ATP _channel subunits) is poorly defined. We examined the localization of each of the K_ATP _channel subunits in the mouse and rat heart.

**Results:**

Immunohistochemistry of cardiac cryosections demonstrate Kir6.1 protein to be expressed in ventricular myocytes, as well as in the smooth muscle and endothelial cells of coronary resistance vessels. Endothelial capillaries also stained positive for Kir6.1 protein. Kir6.2 protein expression was found predominantly in ventricular myocytes and also in endothelial cells, but not in smooth muscle cells. SUR1 subunits are strongly expressed at the sarcolemmal surface of ventricular myocytes (but not in the coronary vasculature), whereas SUR2 protein was found to be localized predominantly in cardiac myocytes and coronary vessels (mostly in smaller vessels). Immunocytochemistry of isolated ventricular myocytes shows co-localization of Kir6.2 and SUR2 proteins in a striated sarcomeric pattern, suggesting t-tubular expression of these proteins. Both Kir6.1 and SUR1 subunits were found to express strongly at the sarcolemma. The role(s) of these subunits in cardiomyocytes remain to be defined and may require a reassessment of the molecular nature of ventricular K_ATP _channels.

**Conclusions:**

Collectively, our data demonstrate unique cellular and subcellular K_ATP _channel subunit expression patterns in the heart. These results suggest distinct roles for K_ATP _channel subunits in diverse cardiac structures.

## Background

ATP-sensitive (K_ATP_) channels are widely expressed in both excitable and non-excitable tissue types throughout the body. However, differences exist in the functional and pharmacological properties of various K_ATP _channels in different tissues. This functional diversity of K_ATP _channels is also reflected in the cardiovascular system. K_ATP _channels are abundantly expressed in ventricular myocytes, where they are probably best characterized. These channels have a high unitary conductance, are inhibited by ATP in the micromolar range, are blocked by glibenclamide (but not tolbutamide) and opened by pinacidil (and not by diazoxide). K_ATP _channels also exist in the coronary vasculature, where they function to maintain basal coronary blood flow [1]. K_ATP _channels in the coronary smooth muscle have a low unitary conductance (~30 pS) and are blocked by glibenclamide and activated by K_ATP _channel openers and adenosine [2]. K_ATP _channels exist in the coronary endothelium [3], but their biophysical properties remain largely unidentified. In addition to this diverse distribution of plasmalemmal K_ATP _channels in the heart, K_ATP _channels with unique biophysical and pharmacological profiles are also believed to be expressed in the mitochondrial inner membrane [4].

K_ATP _channels are increasingly well characterized at the molecular level. In order to express a functional channel that resembles native K_ATP _channels in terms of their biophysical and pharmacological properties, a combination of two types of subunits is necessary. It is now understood that Kir6 subunits form a pore-forming structure through which K^+ ^ions transverse the membrane whereas SUR subunits assemble with the latter to modulate the channel's function and to confer unique pharmacological properties to the channel complex [5,6]. Two genes each code for the two known Kir6 subfamily members (Kir6.1 and Kir6.2) and for the two known SUR members (SUR1 and SUR2). Alternative splicing of SUR2 gives rise to at least two functionally relevant isoforms (SUR2A and SUR2B) with distinct pharmacological profiles [5]. It is widely believed that ventricular K_ATP _channels consist of the specific combination of Kir6.2 and SUR2A subunits and that K_ATP _channels in vascular smooth muscle consist of Kir6.1 and SUR2B subunits. This view is consistent with results from gene targeting experiments, which demonstrate the absence of functional sarcolemmal K_ATP _channels in ventricular myocytes from Kir6.2(-/-) mice and the coronary abnormalities that develop in Kir6.1 and SUR2 null mice [5]. Although they are powerful tools, gene knockout approaches can overemphasize certain important aspects of gene function and may overlook more subtle effects of protein function and interaction. At first sight, these models do not adequately explain the reports of SUR1 mRNA expression in the heart [7], or the observation that anti-SUR1 antisense oligonucleotides inhibit K_ATP _channels of ventricular myocytes [8]. They also do not provide a functional basis for the known expression of Kir6.1 mRNA and protein in cardiac myocytes [9-12]. or explain the molecular composition of the endothelial K_ATP _channel. The specific cellular and subcellular localization of proteins can be used to predict their function. We therefore used antibodies specific for each of the K_ATP _channel subunits to determine their cellular and subcellular localization in the mouse and rat heart. Our results suggest distinct roles for each of the K_ATP _channel subunits in diverse cardiac structures.

## Results

Given the reports of expression of each of the K_ATP _channel subunits in the heart (see earlier), we performed immunohistochemistry and immunocytochemistry to determine the cellular and subcellular localization of Kir6.1, Kir6.2, SUR1 and SUR2 subunits in mouse and rat ventricle. To this end, we stained frozen sections of cardiac ventricular tissue as well as cardiac myocytes enzymatically isolated from mouse and rat hearts. Where possible, we used different antibodies to the subunits to ensure that the staining pattern observed was specific.

### Characterization of the antibodies used in this study

**Figure 1:**
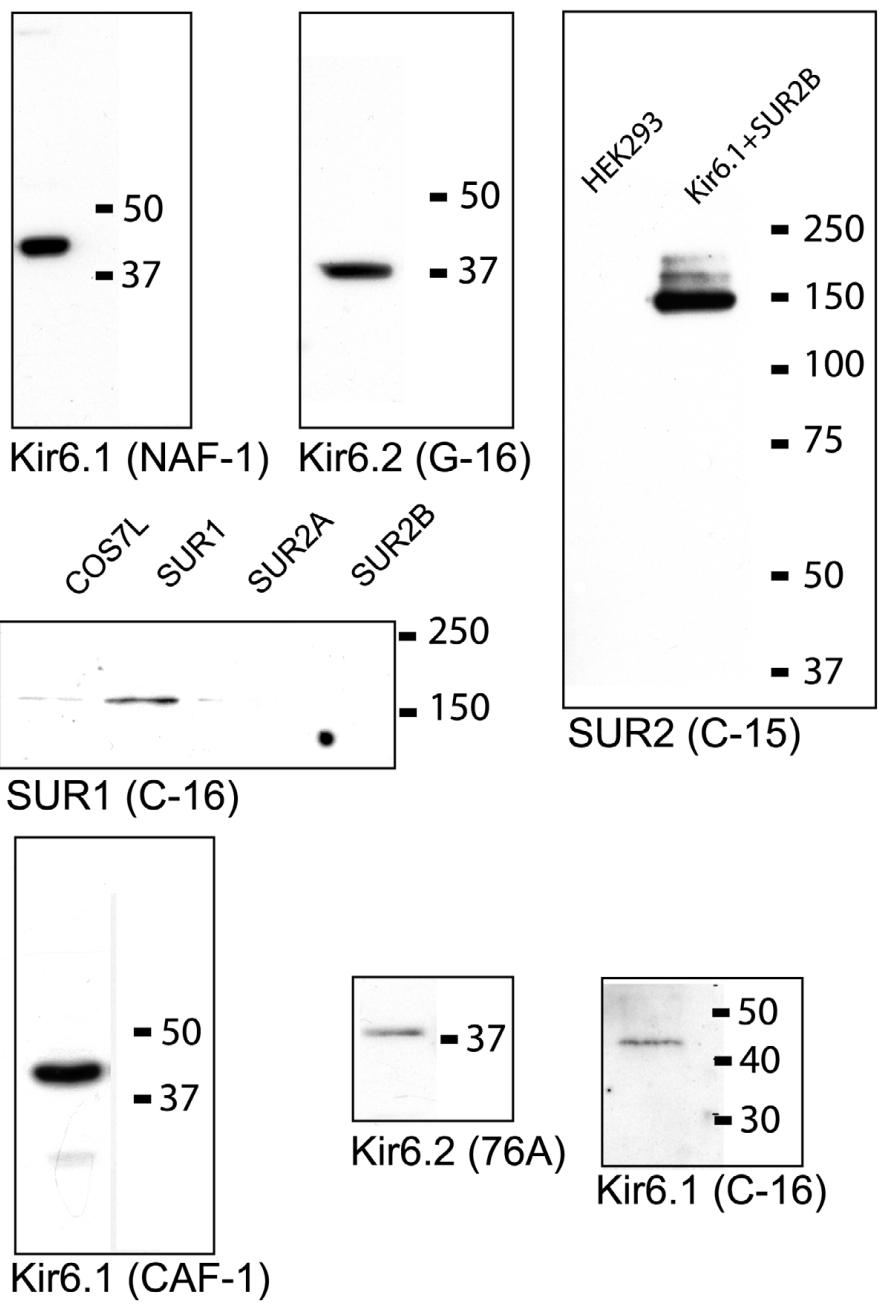
Characterization of the antibodies used in this study. Rat heart membrane fractions were separated on 8–12% denaturing PAGE and Western blotting was performed using Kir6.1 (NAF1, CAF-1 or C-16) or Kir6.2 (G-16 or 76A) antibodies. In other experiments, lysates of HEK293 cells stably transfected with Kir6.1/SUR2B, or lysates of COS7L cells transiently transfected with SUR1, SUR2A or SUR2B cDNAs, were separated with PAGE and respectively immunoblotted with anti-SUR2 (C-15) or anti-SUR1 (C-16) antibodies. Molecular size markers are indicted as appropriate.

We performed Western blotting to determine the specificity of the antibodies used in this study. Three different anti-Kir6.1 antibodies (NAF-1, CAF-1 and C-16) all detect a band that migrates with an apparent molecular size of 44 kDa in Western blotting of rat heart membrane fractions (Fig 1). A 44 kDa band was also detected by the 78A antibody (not shown). These antibodies did not cross-react with Kir6.2, since they detected only Kir6.1 (and not Kir6.2) in parallel experiments on cells transfected with various K_ATP _channel subunit combinations (data not shown, see also reference [13]).

Both the 76A and G-16 anti-Kir6.2 antibodies have previously been characterized and we demonstrated that they specifically detect a ~38 kDa band in Western blotting of Kir6.2 transfected cells and do not detect heterologously expressed Kir6.1 protein [13,14]. Here we show that both of these antibodies also detect Kir6.2 subunits as a ~38 kDa band in Western blotting of heterologously expressed Kir6.2 protein or rat heart membrane fractions (Fig 1).

The anti-SUR1 antibodies specifically detect SUR1 protein (170 kDa) in cell lysates of COS7L cells transiently transfected with SUR1/Kir6.2 cDNA as well as in membrane fractions obtained from mouse hearts [15]. In the cell lystates from SUR2B/Kir6.2 transfected cells, the SUR2 antibody recognizes a specific band at 150 kD in transfected cells only (Fig 1) and did not detect SUR1 (not shown). Thus, each of the antibodies used in this study detected proteins at the correct molecular size in Western blotting and did not cross-react non-specifically with other proteins.

### Kir6.1 localization in the murine heart

**Figure 2:**
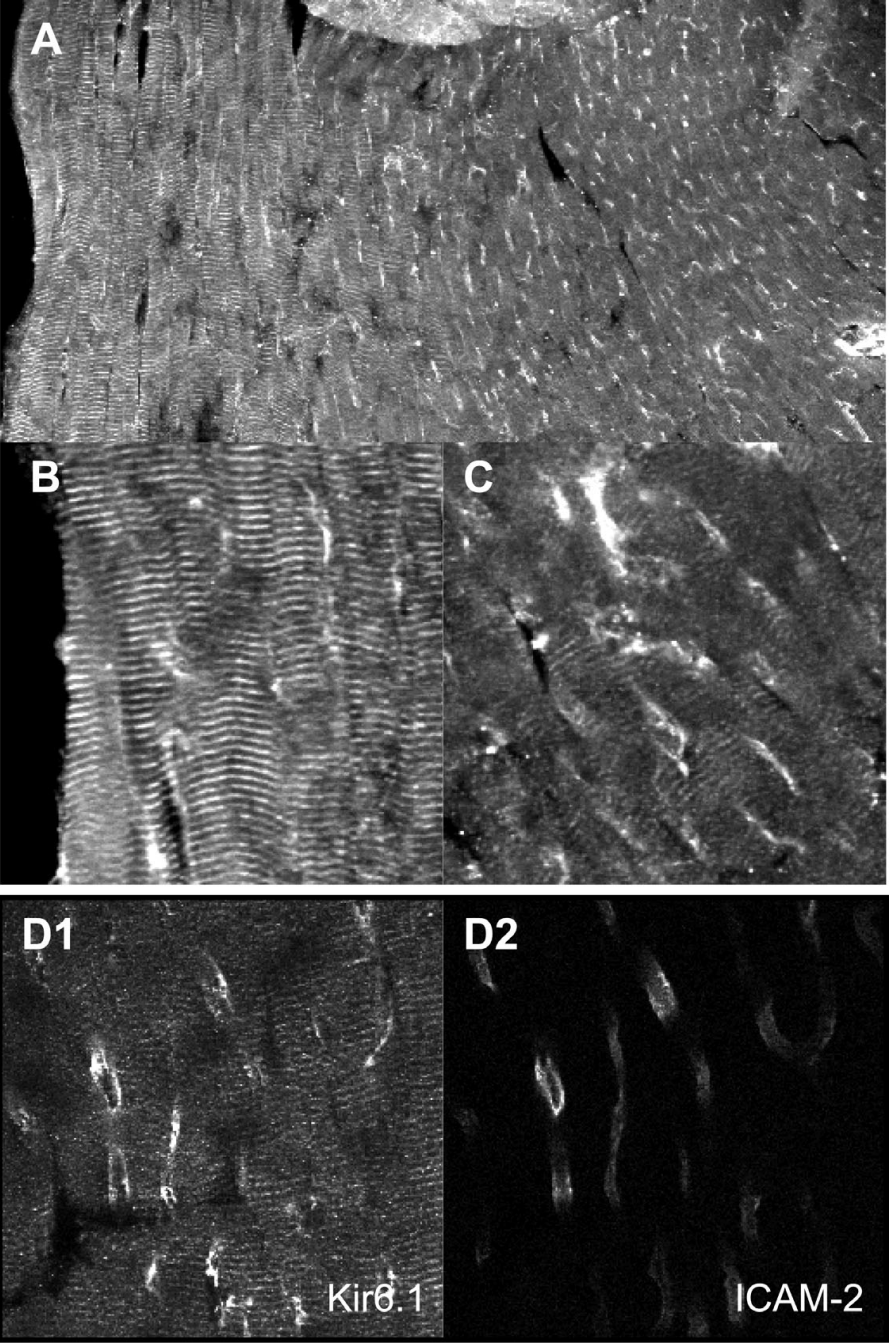
Immunohistochemistry demonstrating the regional distribution of Kir6.1 subunits in cryostat sections of the mouse ventricle. **A**: The low magnification image demonstrates regional expression differences with more prominent myocyte staining apparent in the epicardium (left) than in the mid-myocardium (right). Image width is 750 μm. **B **and **C**: Higher magnification of the same slide shown in previous panel showing an epicardial (B) and midmyocardial section (C). The image widths are respectively 102 and 96 μm. **D**: Double stained section of a midmyocardial section using NAF-1 anti-Kir6.1 antibodies (D1) and ICAM-2 antibodies as a marker of endothelial cells. (D2). The image width is 119 μm. The secondary antibodies used were Cy-3 conjugated donkey anti-rabbit and Cy-5 conjugated donkey anti-rat F(ab')2 IgG fragments.

We used an immunohistochemistry approach to identify the localization of Kir6.1 protein in cryostat sections of mouse ventricles. We used three separate antibodies that produced similar results (NAF-1, CAF-1 and C-16). We also used another anti-Kir6.1 antibody (78A) but this antibody did not perform well in these assays. A typical result is shown in Fig 2A where Kir6.1 protein was ubiquitously detected throughout the ventricle. Closer inspection shows that Kir6.1 protein is expressed in a sarcomeric striated pattern in ventricular myocytes. This effect is more pronounced in epicardial myocytes (left hand side of panel A and Fig 2B). In the midmyocardium, a punctate staining pattern is apparent (Fig 2C). This was observed in over 50 different cryosections that we examined (also observed with the CAF-1 antibody).

**Figure 3:**
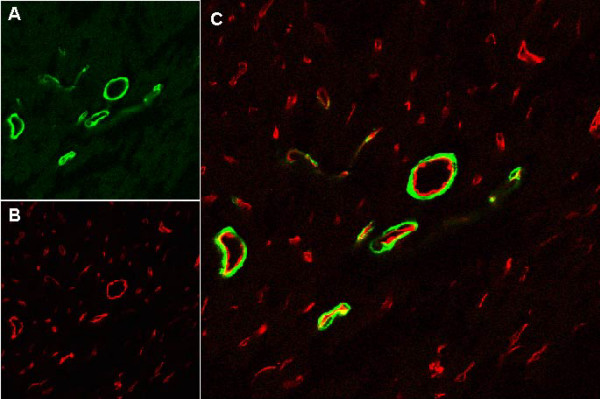
Immunohistochemistry markers to distinguish between vascular smooth muscle and endothelial cells. A cryosection of mouse heart ventricle was simultaneously stained with FITC-conjugated smooth muscle α-actin antibody (A) and an antibody against the apical endothelial membrane protein, ICAM-2 (B). The secondary antibodies to detect ICAM-2 was Cy-5 conjugated donkey anti-rat (pseudo-colored red for clarity). Panel C is an overlay of the preceding two panels. The image width is 238 μm.

The cylindrical shape of the punctate structures in Fig 2C is reminiscent of coronary blood vessels. We tested various antibodies to find suitable markers for smooth muscle- and endothelial cells in the coronary vasculature. We found antibodies against smooth muscle α-actin to be a good marker for coronary vessels (Fig 3A) typically having diameters upwards of about 10 μm. We also tested an antibody against the apical endothelial protein ICAM-2 and found the antibody to detect endothelial cells lining the inner layer of coronary arteries (Fig 3B and 3C). Additionally, the anti-ICAM-2 antibodies stained a large number of smaller vessel-like structures having diameters typically smaller than 15 μm (average around 6–8 μm). These smaller vessels apparently do not have an appreciable amount of vascular smooth muscle cells, as judged by the lack of smooth muscle α-actin staining, suggesting that they may be coronary capillaries (the possibility that some of them may be pre-capillary arterioles with low amounts of smooth muscle cannot be excluded). To examine the localization of Kir6.1 in coronary blood vessels, we co-stained mouse ventricle with NAF-1 anti-Kir6.1 antibodies and anti-ICAM-2 antibodies (a marker for the vascular endothelium). As shown in Figure 2D, there is clear correspondence between Kir6.1 staining and ICAM-2 localization, suggesting high expression levels of Kir6.1 in the coronary vasculature.

**Figure 4:**
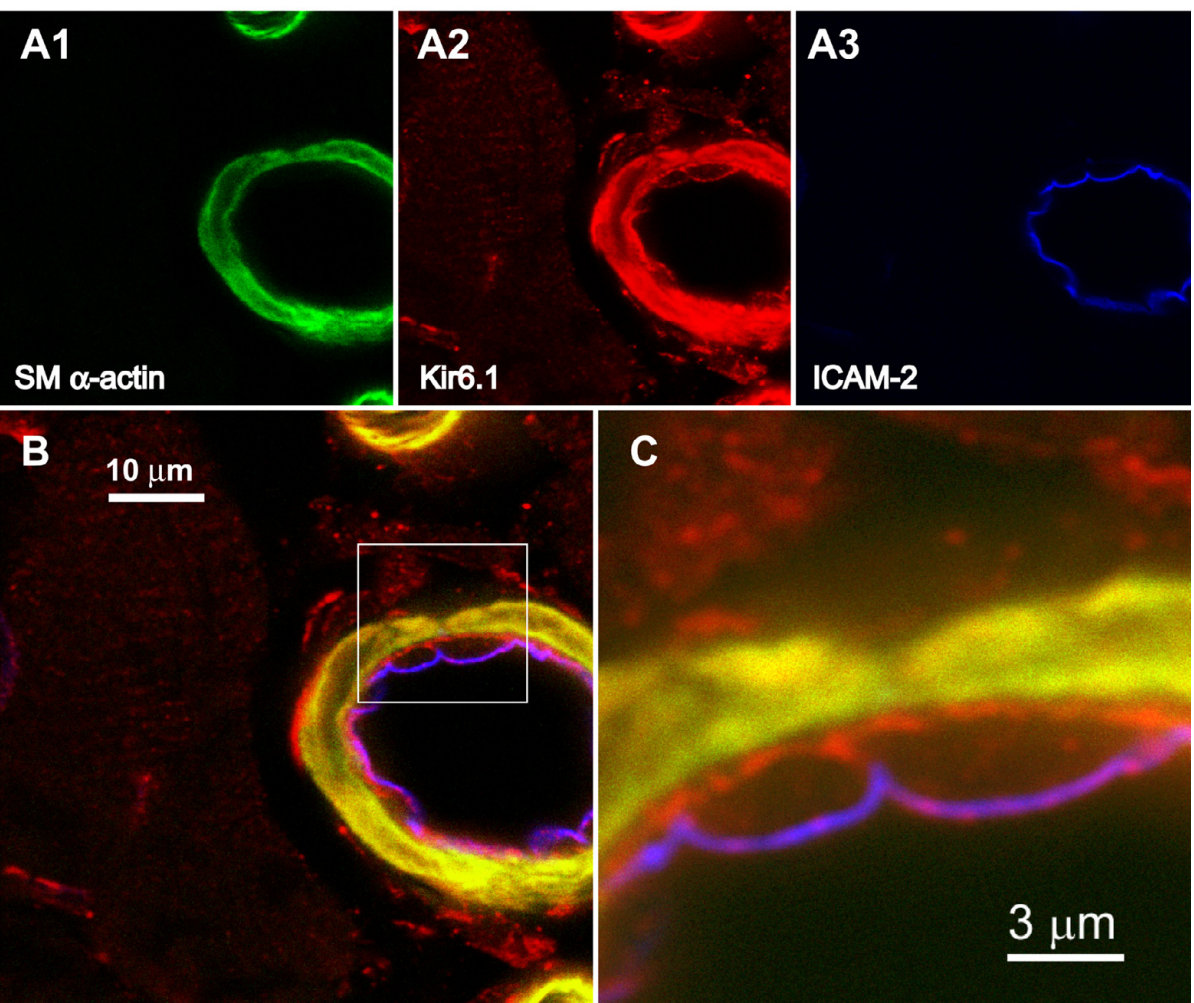
Triple stain immunohistochemistry of mouse ventricle demonstrating the distribution of Kir6.1 subunits in the coronary vasculature. The cryostat section was stained with antibodies against smooth muscle α-actin (**A1**), Kir6.1 (**A2**) and endothelial-specific ICAM-2 (**A3**). **B**:Overlay of the three panels shown in panels A. **C**: Higher magnification of the boxed area highlighted in panel B.

Given the high sensitivity of the NAF-1 antibody, we were able to perform a sub-cellular localization study of Kir6.1 protein in the mouse heart (Fig 4; this approach was not possible with other antibodies, which produced less sensitive staining). We co-stained a cryostat section with antibodies against smooth muscle α-actin (A1), Kir6.1 (A2) and the endothelial ICAM-2 protein (A3). An overlay of these three images is shown in panel B. A higher magnification of the area roughly represented by the boxed area (panel C) demonstrates that Kir6.1 subunits are ubiquitously expressed and are present in ventricular myocytes, the coronary smooth muscle walls as well as in endothelial cells. Of these cells types, the highest expression levels appear to occur in the vasculature.

### Kir6.2 localization in the murine heart

**Figure 5:**
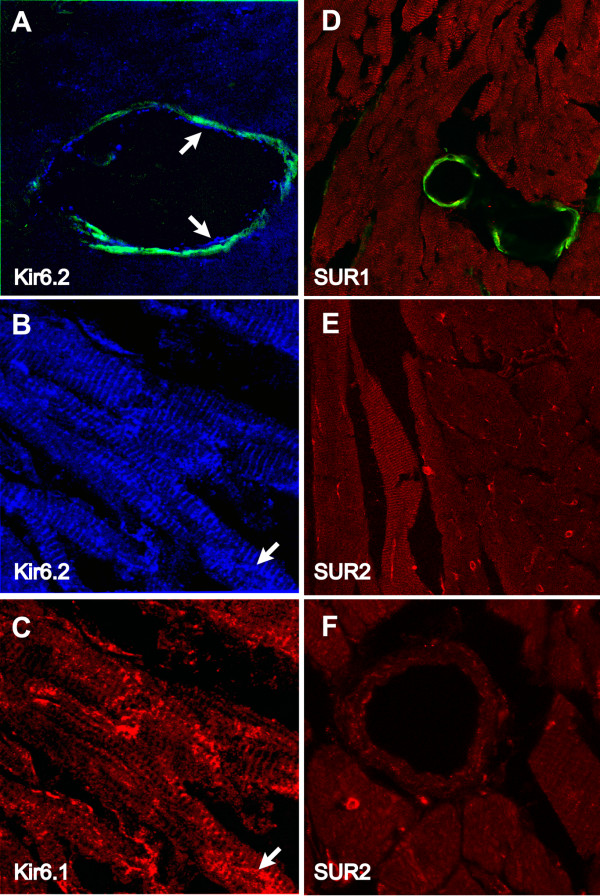
Immunohistochemistry demonstrating the cellular distribution of the Kir6.2, SUR1 and SUR2 subunits in cryostat sections of the mouse ventricle. **A**: Co-staining of mouse ventricular section with smooth-muscle α-actin (green) and anti-Kir6.2 antibodies (Santa Cruz). The latter antibody was detected with Cy-5 conjugated donkey anti-goat IgG (blue). **B and C**: Double-staining with anti-Kir6.2 antibodies (Santa Cruz) and CAF-1 anti-Kir6.1 antibodies (detected with Cy-3 conjugated donkey anti-chicken IgY (red). The image width of panel A is 109 μm and for panels B and C it is 89 μm. **D**: Co-staining of a midmyocardial section with anti-SUR1 antibodies (Cy3-conjugated donkey anti-goat secondary antibodies were used; shown in red) and FITC-conjugated smooth muscle anti-actin antibodies (shown in green). **E **and **F**: Cryosections stained with a pan anti-SUR2 antibody (secondary antibody was Cy-3 conjugated anti goat IgG. The image widths for panels D-F respectively are 238, 238 and 88 μm.

We used several antibodies against Kir6.2 subunits to determine their cellular localization. Both the 76A and the G-16 antibodies (Santa Cruz) gave similar results. The W62 antibody developed by us [13] did not seem to stain more than background and we therefore assumed this antibody not to work in this assay. In terms of staining in the vasculature, we detected Kir6.2 subunit expression mainly in the endothelium (arrows in Fig 5A) and not in smooth muscle (as judged by a lack of co-localization with smooth muscle α-actin). Evidently, Kir6.2 subunits were expressed in cardiac myocytes as well, as demonstrated in Fig 5B. The staining occurs in a sarcomeric striated pattern in ventricular myocytes (distance interval of roughly 2.2 μm). Although not as apparent as with Kir6.1, there appears to be expression of Kir6.2 subunits in small coronary blood vessels (10 μm or less). These structures are also stained by Kir6.1 antibodies (arrows in Fig 5B and 5C), suggesting co-localization of Kir6.1 and Kir6.2 subunits in small coronary blood vessels.

### Localization of SUR subunits in murine heart

Using anti-SUR1 antibodies, we observed staining predominantly in cardiac ventricular myocytes (Fig 5D). The lack of staining of either large or small coronaries suggests that SUR1 subunits are not expressed in the coronary vasculature of the mouse heart (note the lack of co-localization of SUR1 with smooth muscle α-actin). In contrast, pan-SUR2 antibodies stain both ventricular myocytes in a regular striated sarcomeric pattern as well as small coronary blood vessels (Fig 5E and 5F). Note the lack of high expression levels in larger coronary arteries (round structure in Fig 5F), demonstrating that expression of SUR2 subunits occurs predominantly in small coronary vessels (less than 10 μm). In separate experiments, we did not detect a particularly strong co-localization with smooth muscle α-actin (not shown), ruling against the possibility that SUR2 is strongly expressed in the smooth muscle cells of larger coronary vessels.

### Subcellular localization of K_ATP _channel subunits in enzymatically isolated ventricular myocytes

**Figure 6:**
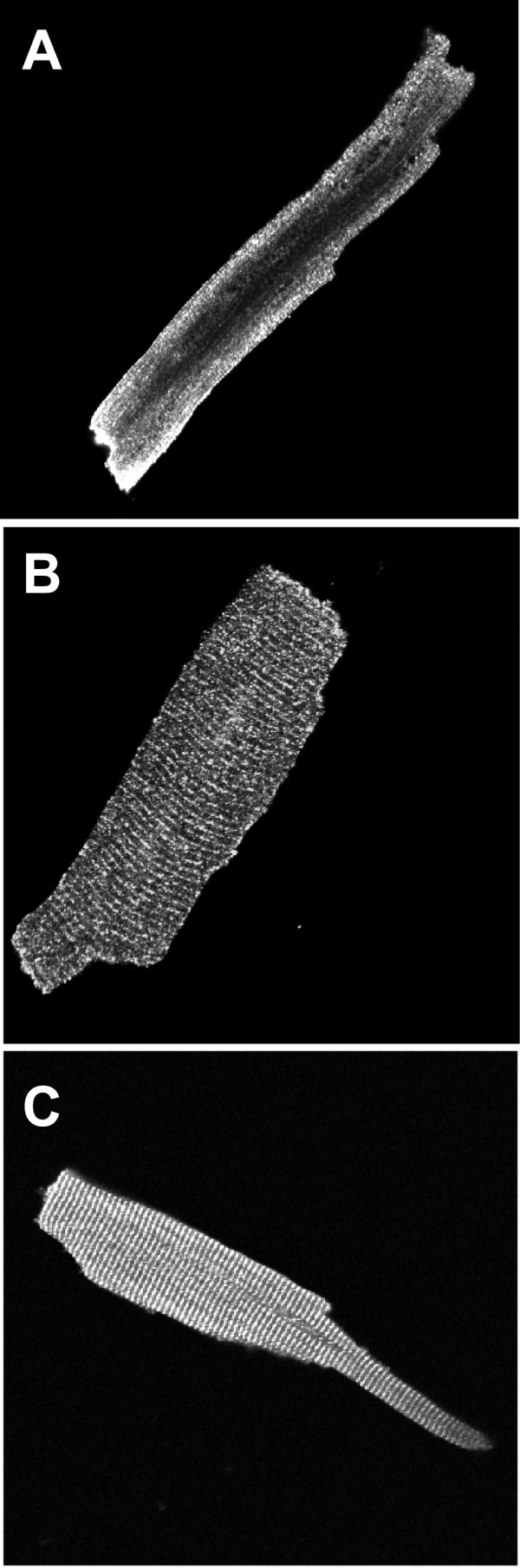
Effect of different fixation protocols on immunocytochemistry of ventricular myocytes. Enzymatically isolated ventricular myocytes were subjected to fixation protocols with (A) methanol, (B) paraformaldehyde or (C) sequential double fixation with paraformaldehyde followed by methanol. The primary antibody in all cases was against Kir6.2 subunits (76A) and the secondary antibody was Cy-3 conjugated donkey anti-rabbit IgG. The image widths are respectively 137, 107 and 139 μm.

Fixation and permeabilization procedures can affect the outcome of immunocytochemistry experiments. We therefore compared three different methods. Isolated cardiac myocytes were (a) fixed with paraformaldehyde and permeabilized with Triton X-100, (b) fixed and permeablized in a single step by ice-cold methanol or (c) subjected to a two-step protocol with paraformaldehyde fixation followed by methanol fixation/permeabilization. Typical results obtained with the anti-Kir6.2 (76A) antibody are shown in Fig 6. In general, we found methanol fixation to preserve membrane staining but to cause a loss of intracellular fluorescence (Fig 6A). Paraformaldehyde fixation, in contrast, better preserved staining of intracellular structures (Fig 6B). The two-step fixation protocol in general gave similar results to paraformaldehyde fixation, but the fluorescence intensity was generally much higher and more clearly defined (Fig 6C). This may be due to better preservation of protein antigenicity and/or improved permeabilization (and hence improved antibody accessibility). Our data are consistent with a recent report describing the two-step protocol to better preserve the in-vivo subcellular localization of proteins [16]. We consequently used the two-step protocol for all subsequent experiments to examine the subcellular localization of K_ATP _channel subunits.

**Figure 7:**
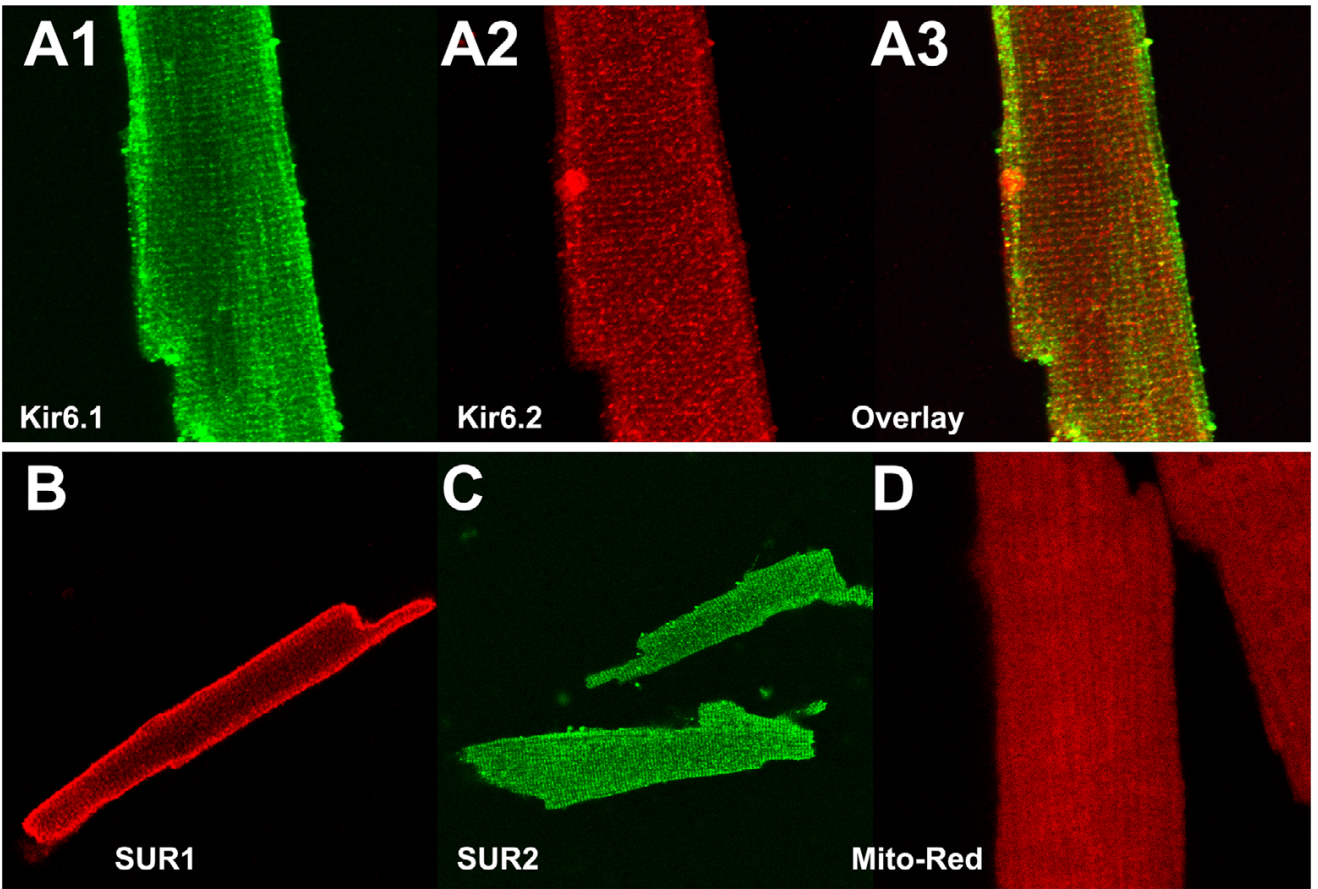
Immunocytochemistry of isolated mouse ventricular myocytes demonstrating the subcellular localization of Kir6.1, Kir6.2, SUR1 and SUR2 subunits. **A**: Double staining of a ventricular myocyte with the CAF-1 anti-Kir6.1 antibody (A1) and 76A anti-Kir6.2 antibody (A2). Panel A3 is an overlay of panels A1 and A2. Secondary antibodies used were Cy-3 conjugated donkey anti-chicken IgY (red) and Cy-2 conjugated donkey anti-rabbit IgG (green). Yellow in panel C demonstrates areas of co-localization. The image width is 91 μm. **B**: Ventricular myocyte probed with anti-SUR1 antibodies and detected with Cy-3 conjugated donkey anti goat secondary antibodies. Image width is 148 μm. **C**: Staining with a pan-SUR2 antibody (detected with Cy-2 conjugated donkey anti-goat IgG). The image width is 229 μm. **D**: An isolated myocyte was stained with MitoTracker Red (500 nM) before being paraformaldehyde fixed and viewed with confocal microscopy Image width is 47 μm.

The subcellular distribution of Kir6.1 and Kir6.2 subunits in isolated mouse ventricular myocytes is shown in Fig 7A. Kir6.2 subunits are expressed in a regular striated pattern throughout the myocyte (A2). Kir6.1 subunits showed a similar expression pattern (A1), with the exceptions that (a) Kir6.1 expression appears to be more punctate and (b) staining is more prominent at the myocardial surface. Since we used antibodies developed in different species (chicken anti-Kir6.1 antibody and rabbit anti-Kir6.2 antibody) we were able to detect both proteins in the same myocyte with little cross-reactivity of the secondary antibodies. Although there is some degree of overlap in the subcellular expression of Kir6.1 and Kir6.2 subunits (yellow in A3), it is apparent that there are areas within the cell that express Kir6.2 but not Kir6.1 subunits. Thus, the possibility exists that these subunits may have different functional roles within the ventricular myocyte. Similarly, SUR1 and SUR2 subunits also have distinct subcellular localizations. We consistently observed strong surface staining with SUR1 antibodies (Fig 7B) whereas SUR2 antibodies diffusely stain throughout the width of the cell in a sarcomeric repeating pattern (Fig 7C). To address the question whether there is co-localization of any of the K_ATP _channel subunits with mitochondria, we used MitoTracker Red as a mitochondrial marker. We found MitoTracker staining to dissipate during immunocytochemistry protocols and co-labeling with K_ATP _channel antibodies was therefore not an effective strategy. Nevertheless, the pattern of MitoTracker staining that we observed shortly after fixation (Fig 7D) was inconsistent with predominant subcellular distribution of any of the K_ATP _channel subunits and we conclude therefore that K_ATP _channel subunits are not abundantly expressed in cardiac mitochondria.

**Figure 8:**
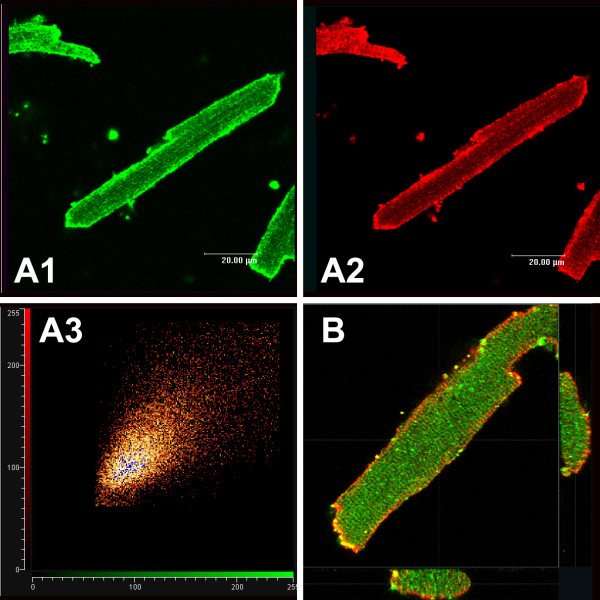
Subcellular localization of Kir6.1, Kir6.2 and SUR1 subunits in ventricular myocytes. **A**: Co-staining of a mouse ventricular myocyte with antibodies to Kir6.1 (A1: NAF-1) or SUR1 (A2: C-16). The secondary antibodies were Cy2-conjugated donkey anti-rabbit and Cy3-conjugated donkey anti-goat. The degree of co-localization after background elimination is depicted in panel A3 by plotting pixel intensities of the two channels against each other. Pseudo-coloring of pixels indicates degree of co-localization (black is low and white high). **B**: Co-staining of a mouse cardiac myocyte with anti-Kir6.2 antibodies (76A) and anti SUR1 antibodies. Secondary antibodies used were Cy-2 conjugated donkey anti-rabbit to detect Kir6.2 (green) and Cy-3 conjugated donkey anti-goat IgG to detect SUR1 (red). A 3-D reconstruction was performed (58 stacked images with a voxel size of 0.23 μm^3^). The image width is 119 μm. The images shown on the bottom and right insets are cut-through projections of the cell width (the regions of cross-sections areas shown by the white lines; the middle of each image is also indicated by a white line for clarity).

Experiments were designed closer to examine the subcellular expression of SUR1 subunits relative to Kir6 pore-forming subunits (Fig 8). There was a remarkable degree of co-localization of SUR1 subunits with Kir6.1 subunits (Fig 8A) but not with Kir6.2 subunits (Fig 8B).

**Figure 9:**
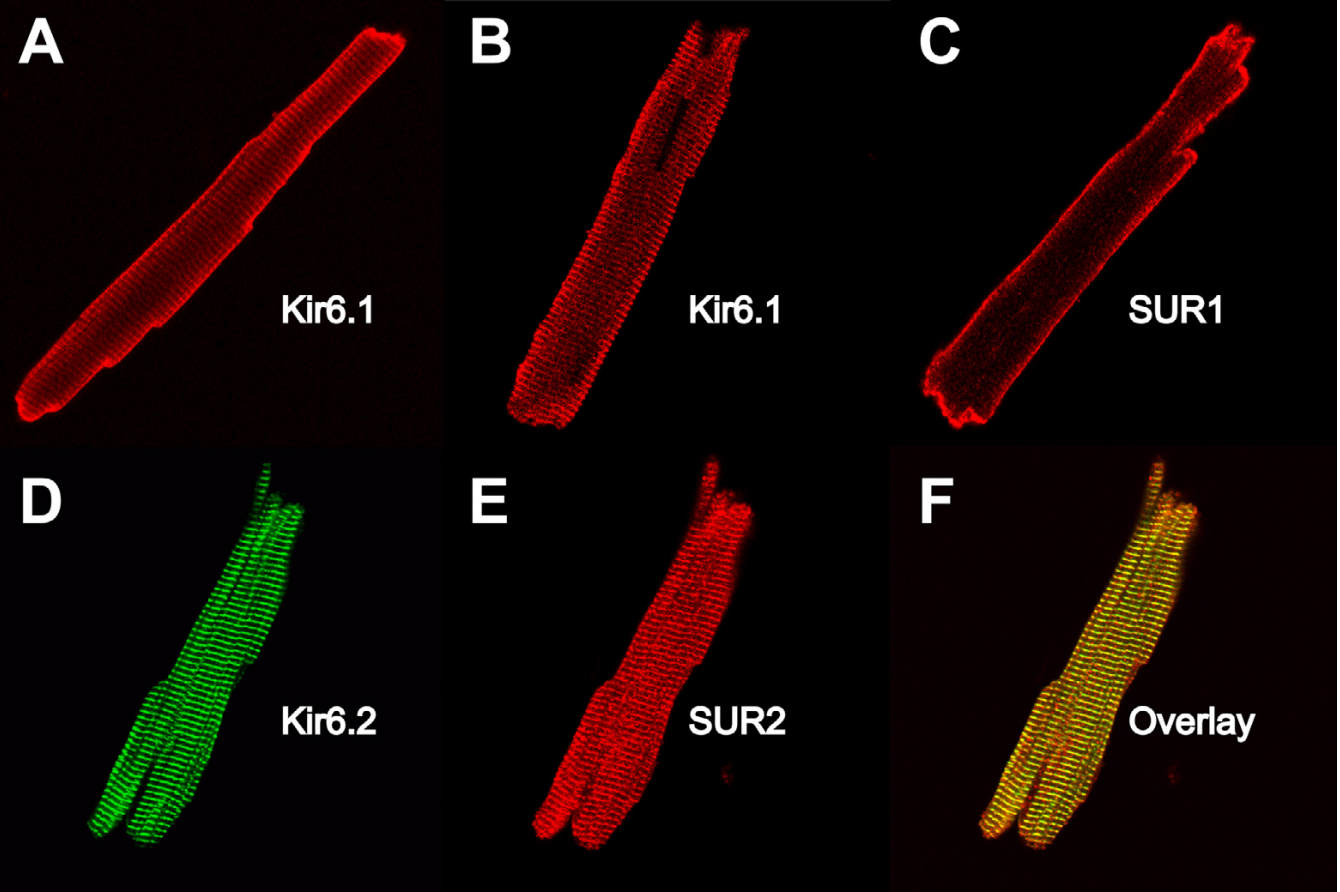
Subcellular localization of K_ATP _channel subunits in isolated rat ventricular myocytes. **A**: Staining with NAF-1 anti-Kir6.1 antibodies (detection with Cy-3 conjugated donkey anti rabbit). Image width is 128 μm. **B**: Staining with a different anti-Kir6.1 antibody (C16, Santa Cruz); secondary antibody used was Cy-3 conjugated donkey anti-goat IgG. Image width is 136 μm. **C**: Staining with an anti-SUR1 antibody. Image width is 128 μm. **D**: Co-localization of Kir6.2 and SUR2 subunits. The preparation was co-stained with an anti-Kir6.2 antibody (76A; detection with Cy-2 conjugated donkey anti-rabbit IgG; green) and SUR2 antibodies (detected with Cy-5 conjugated donkey anti-goat IgG; pseudo-colored red for clarity). Panel D3 is an overlay of panels D1 and D2; yellow is indicative of co-localization. Image width is 125 μm.

We finally examined the subcellular expression patterns of K_ATP _channel subunits in another species. We chose rat ventricular myocytes for this purpose. We essentially obtained the same results as with mouse myocytes. Using different antibodies against Kir6.1 (NAF-1 and C16) we observed strong surface expression of this subunit with a smaller degree of intracellular labeling (Fig 9A and 9B). SUR1 antibodies also strongly labeled the cell surface (Fig 9C). In contrast, Kir6.2 and SUR2 antibodies both labeled repetitive patterns at sarcomeric distances (Fig 9D and 9E). Furthermore, there is strong co-localization of Kir6.2 and SUR2 subunits in ventricular myocytes (yellow in Fig 9F).

## Discussion

### Antibodies used in this study

A significant strength of our study is that we extensively characterized the antibodies used. We performed Western blotting with membrane fractions obtained from the heart to ensure that a band of the expected size is detected. Further, we chose antibodies that showed little cross-reactivity with other proteins, as judged by the absence of non-specific bands. In as far as it was possible we used different antibodies to the same K_ATP _channel subunits in immunostaining experiments to ensure that the same cellular and subcellular distribution staining patterns occurred. Although not shown, we always performed negative controls to ensure that no staining was observed when the primary antibodies were omitted (to eliminate non-specific staining by the secondary antibodies used) or that staining could be blocked by preincubation of antibody with the peptide to which the antibody has been raised. Further, we used the primary antibodies at the lowest concentrations possible to eliminate possible non-specific cross-reactivity with other proteins. Our study is a comprehensive description of the cellular and subcellular expression patterns of K_ATP _channel subunits in the heart given our stringent criteria and the panel of antibodies available to us.

### Expression of Kir6.2 and SUR2 subunits in ventricular myocytes

Sarcolemmal K_ATP _channels in ventricular myocytes have been described more than two decades ago [17]. Cardiac sarcolemmal K_ATP _channels have been described to consist of hetero-octameric complexes of Kir6.2 and SUR2A subunits [5,18,19]. This concept was based on the similarities in the biophysical and pharmacological characteristics when comparing heterologously expressed Kir6.2/SUR2A channels with native cardiac K_ATP _channels [20] and also because of the known expression of Kir6.2 and SUR2A mRNA and protein in the heart. Our data demonstrate both Kir6.2 and SUR2A subunits to be expressed in ventricular myocytes. Furthermore, we find that these two subunits co-localize, which is consistent with the biochemical, functional and pharmacological data supporting the concept that they can combine to form a heteromeric channel complex [5]. Our data are also in agreement with the finding that knockout mice deficient of Kir6.2 or SUR2 subunits lack K_ATP _channels in the ventricular myocyte [21,22]. It is interesting that Kir6.2/SUR2 subunits are expressed in a regular striated pattern in ventricular myocytes. Furthermore, close inspection of the images shows that both Kir6.2 and SUR2 expression is somewhat punctate. These observations are in complete agreement with previous studies describing the expression of SUR2 isoforms in the t-tubules and sarcolemma [23] and the subcellular localization of sarcolemmal K_ATP _channels as determined by functional microscopy. Scanning ion conductance (patch clamp) microscopy data have demonstrated K_ATP _channels to be organized in small clusters and to be anchored in the Z-grooves (t-tubular openings) of the sarcolemma [24]. Collectively, these data suggest that K_ATP _channels are predominantly expressed in the t-tubular system. The implications of K_ATP _channels present in the t-tubular system are not entirely clear. Since t-tubular ion channels may control action potential propagation into the cardiac myocyte, it may be possible for K_ATP _channels to have a role in the spread of excitation and action potential duration, particularly during conditions of metabolic impairment when these channels are more prone to opening. A shorter action potential duration in the t-tubular system would imply less Ca^2+ ^entry at the local control sites of SR Ca^2+ ^release and hence reduced contractility, which may in part explain the negative inotropic effects observed with K_ATP _channel openers [25]. However, the picture may be more complex since both Kir6.1 and SUR1 subunits are also expressed in ventricular myocytes.

### Expression of Kir6.1 and SUR1 subunits in ventricular myocytes

We found clear expression of Kir6.1 and SUR1 subunits in cardiac ventricular myocytes. Interestingly, both of these two subunits are strongly expressed at the sarcolemmal surface, but their functions in the sarcolemma are currently not understood. Since ventricular K_ATP _channels can be recorded in hearts from knockout mice lacking Kir6.1 subunits [9], it appears that Kir6.1 subunits are not an absolute requirement for the formation of functional ventricular K_ATP _channels. It may be possible for Kir6.1 subunits to have a role in the pathophysiological setting, as demonstrated by the upregulated Kir6.1 expression levels during cardiac remodeling after ischemia or hypoxia [11,26]. To our knowledge, cardiac K_ATP _channels have not been studied in SUR-/- mice. However, SUR1 antisense oligonucleotides inhibit K_ATP _channels in rat ventricular myocytes [8], suggesting a functional role for these subunits in ventricular sarcolemmal K_ATP _channel function. Our data, demonstrating that both Kir6.1 and SUR1 subunits exhibit strong sarcolemmal expression, may require a reassessment of the molecular composition of ventricular K_ATP _channels during normal and pathophysiological conditions.

### Expression of Kir6 and SUR subunits in mitochondria

The concept has evolved that K_ATP _channels are expressed in the mitochondrial inner membrane and that these channels are involved in the protection of the heart afforded by ischemic preconditioning [27,28]. The molecular nature of mito-K_ATP _channels remains to be identified. There are almost as many reports describing the presence of Kir6.0 subunits in cardiac mitochondria [23,29,30]. as there are denouncing their existence in these organelles [31,32]. We did not observe strong localization of K_ATP _channel subunits in ventricular mitochondria. However, the technique of immunocytochemistry does not have sufficient resolution to rule out the existence of K_ATP _channel subunits in mitochondria and our data therefore do not add significantly to this debate, other than demonstrating that K_ATP _channel subunits are not abundantly expressed in mitochondria of ventricular myocytes.

### Expression of K_ATP _channel subunits in the coronary smooth muscle

A tight coupling exists between metabolic status in the heart and coronary blood flow. K_ATP _channels have been identified in several different vascular tissues, including the coronary vasculature [33]. K_ATP _channels in coronary resistance vessels have also been implicated in physiologically important stimuli such as regulation of basal vascular tone, autoregulation of blood flow, hypoxia-induced coronary vasodilation, reactive hyperemia (a clinical index of coronary reserve) and ischemia (reviewed in [33,34]).

Molecularly, the identity of coronary smooth muscle K_ATP _channels has been characterized less extensively than the K_ATP _channels in cardiomyocytes. A recent study employing in situ hybridization histochemistry examined Kir6.1 and SUR2B mRNA expression in different vascular beds, including the coronary vasculature [35]. Strong mRNA expression of these two subunits was found in coronary resistance arteries. Interestingly, Kir6.1/SUR2B expression was not found in coronary veins or venules. We found expression of Kir6.1 and SUR2B protein in primary human coronary artery smooth muscle cells and cryosections of human ventricle [13]. The present study is the first systematic and comparative characterization of K_ATP _channel subunit expression in the intact coronary vasculature. We find Kir6.1 expression in blood vessels of different sizes, including large vessels such as the aorta and large arteries, but also in small resistance arterioles (as defined by their diameter of larger than 12–15 μm and the presence of a well-defined smooth muscle layer). Without using specific markers, we were not able to distinguish objectively between venules and arterioles, but we did occasionally observe vessels with a thin smooth muscle layer (possibly veins or venules) that only expressed Kir6.1 faintly (if at all). Collectively, our data using various anti-Kir6.1 antibodies generally suggest that Kir6.1 subunits are expressed in coronary arterial smooth muscle, and possibly to a lesser extent in coronary veins. In contrast, we did not observe any staining of the coronary smooth muscle with anti-Kir6.2 antibodies.

We found strong staining of smaller coronary resistance vessels with the anti-SUR2 antibodies. We did not have access to suitable SUR2 isoform-specific antibodies, but the staining most probably reflects SUR2B expression. Curiously, we failed to see strong SUR2 expression in larger coronary arteries. This result is in apparent contradiction to the description that SUR2B mRNA expression occurs in larger coronaries [35] and the lack of K_ATP _channels in the aortic cells of the SUR2 knockout mouse [36]. Reasons for this discrepancy are unclear, but may relate to the lack of sensitivity of the anti-SUR2 antibodies used, thus underestimating SUR2 protein expression in other structures. We did not observe SUR1 protein expression in the coronary smooth muscle. Our data are therefore in full support of the notion that K_ATP _channels in coronary artery smooth muscle (particularly the smaller resistance vessels) are comprised of Kir6.1/SUR2 subunits (most likely the SUR2B isoform).

### Expression of K_ATP _channel subunits in the coronary endothelium

The evidence that endothelial K_ATP _channels play a role in regulation of coronary blood flow is compelling. Endothelial K_ATP _channels, for example, contribute to shear stress-induced endothelial release of the vasodilator nitric oxide in rabbit aorta [37] and may also mediate vasodilation in response to hyperosmolarity or acidosis in the coronary microvasculature [38,39]. Furthermore, the powerful vasodilatory effect of adenosine may also be mediated (at least in part) by endothelial K_ATP _channels by stimulating the release of nitric oxide from the endothelium [40].

In the present study, we used immunohistochemistry approaches and identified Kir6.1, Kir6.2 and SUR2 protein in the endothelium lining coronary vessels (Fig. 4) as well as in coronary capillary endothelium (as defined by their small size of less than 10 μm, the presence of the endothelial marker ICAM-2 and the absence of vascular smooth muscle). Our data are supported by previous studies. Using RT-PCR techniques, it has been established that guinea pig coronary endothelial cells express Kir6.1, Kir6.2 and SUR2B subunits [41]. The presence of Kir6.1 and SUR2B mRNA also detected in the coronary endothelium using in situ hybridization histochemistry techniques [35]. Recently, using a combination of RT-PCR and Western blotting techniques, we also identified Kir6.1, Kir6.2 and SUR2B mRNA and protein expression in primary human coronary artery endothelial cells [13]. Importantly, in the latter study we used co-immunoprecipitation approaches to demonstrate that native human coronary endothelial K_ATP _channels are heteromeric Kir6.1/Kir6.2 complexes in combination with SUR2B subunits [13]. Thus, the biophysical nature, modes of regulation and functional consequences of these heteromeric K_ATP _channels in the endothelium may differ fundamentally from homomeric K_ATP _channels found in other tissues. The investigation of endothelial K_ATP _channels is currently the subject of some of our ongoing studies.

### Reservations

For this type of study, the specificity of antibodies used is always a concern. To overcome this problem, we used multiple different antibodies where possible and obtained similar staining patterns. However, we only had access to a single antibody to each of the SUR subunits and consequently we have not been able to verify the specificity of these antibodies by comparing different antibodies to each other (as we have done in the case of the Kir6 subunits). Furthermore, we did not have access to antibodies to the various splice variants of SUR1 or SUR2 and our data therefore do not address the possibility of regional expression differences. A definitive study will require the use of tissues obtained from knockout animals (i.e. the immunostaining should be unequivocally absent in tissues from knockout animals). Viable knockout animals for each of the proteins under consideration have been generated, but we have not been able to obtain these animals (or tissues from these animals) for this purpose. Therefore, although we have taken every step possible to minimize non-specificity issues, our results should be interpreted within this limitation.

## Conclusions

Our study is a comprehensive analysis of the various K_ATP _channel subunits in the heart. We found each of the K_ATP _channel subunits to be expressed in ventricular myocytes, but with varying expression patterns. The roles of Kir6.1 and SUR1 subunits in ventricular myocytes remain to be elucidated and may require a reassessment of the molecular nature of the cardiac K_ATP _channel. Coronary smooth muscle expresses predominantly Kir6.1 and SUR2 subunits, whereas the coronary endothelium expresses Kir6.1, Kir6.2 and SUR2 subunits. Thus, there is wide diversity of K_ATP _channel subunit expression within the heart which determines the functional responses of various cell types to physiological and pathophysiological demands.

## Methods

### Immunohistochemistry

Adult mice were sacrificed by pentobarbital overdose and the hearts were rapidly removed. All animal experiments were approved by the institutional Animal Care Review Board. Hearts were perfused at 37°C through the aorta (Langendorff mode) with Tyrode's solution (in mM: NaCl 137, KCl 5.4, HEPES 10, CaCl_2 _1.8, MgCl_2 _1, NaH_2_PO_4 _0.33; pH adjusted to 7.4 with NaOH) containing pinacidil (100 μM) to cause maximal vasodilatation as to clear the vasculature of blood. Hearts were fixed by switching the perfusate to paraformaldehyde (4% in phosphate-buffered saline (PBS), pH adjusted to 7.4) for 15 minutes at room temperature. The heart was incubated in 4% paraformaldehyde overnight at 4°C. Following fixation, the tissue was incubated overnight at 4°C in 30% sucrose in PBS. The tissue was then embedded in M1 embedding matrix (Thermo Shandon, Pittsburgh, PA) and placed on dry ice until frozen. The block containing the tissue was sectioned using a cooled (-20°C) cryostat (Microm Cryo-Star HM 560, Kalamazoo MI) at 15 μm thicknesses. The sections were transferred to Superfrost Plus slides (Fisher Scientific) for further processing.

Tissue sections were allowed to warm to room temperature. The staining protocol was carried out in a moist chamber to avoid dehydration. Blocking was performed for 60 min with Tris-buffered saline (TBS; in mM 137 NaCl, 50 Tris, 2.7 KCl, pH 7.4) containing 4% goat or donkey serum (depending on the secondary antibody being used) and 0.2% Triton X-100 at room temperature. The slides were incubated overnight at 4°C with primary antibodies (see below) diluted in TBS containing 0.1% serum and 0.2% Triton X-100. Double or triple immunofluorescent studies were carried out by incubating the tissue sections with more than one primary antibody at the same time. Sections were washed three times for 15 min each in TBS, and incubated with fluorescently labeled secondary antibodies (see below) for 1 h at room temperature. The samples were again washed three times for 15 min each in TBS. Sections were drained by blotting with filter paper and a drop of mounting medium (containing an anti-fade reagent) was added to the slides before mounting with a standard coverslip. The mounting medium was allowed to dry before the slides were imaged using a Leica PS2 confocal microscope equipped with an Argon 488 nm gas laser and Helium Neon lasers (543 and 633 lines). Most images were obtained using an emission pin hole of 1.1–1.6 AE with either a 20× (0.7 NA) or a 63× (1.2 NA) oil objective.

### Isolation and immunocytochemistry of cardiac myocytes

Single ventricular myocytes were isolated from adult rats or mice using previously described procedures. Briefly, adult animals were sacrificed and the heart was rapidly removed and perfused in Langendorff mode (at 37°C) for sequential 5 min periods with Tyrode's solution and nominally Ca^2+^-free Tyrode's solution (same composition, but without the addition of CaCl_2_). Myocytes were dispersed using collagenese (Sigma type I; Sigma-Aldrich Chemical Corp, St. Louis, MO, USA) and proteinase (Sigma type XXIV). The ventricles were removed and chopped into small pieces and digested using Ca^2+^-free Tyrode's solution containing the same enzymes. Single dissociated myocytes were plated onto laminin (10 ug/cm^3^)-coated glass coverslips and incubated at 37°C for 15 min to allow attachment to the coverslips before being fixed. In some experiments, myocytes were incubated with 500 nM MitoTracker Red 580 (Molecular Probes, Eugene, OR) during this period.

Three different fixation protocols were employed. Some myocytes were fixed in paraformaldehyde (4%) for 15 min at room temperature, whereas with others fixation and permeabilization was performed in a single step by incubation with ice-cold 100% methanol for 5 minutes at -20°C. However, in the majority of the myocytes presented in this study a two-step fixation protocol was used [16], in which myocytes were first fixed with paraformaldehyde (as described above) followed by methanol fixation/permeabilization. Irrespective of the fixation method, myocytes were then washed with Ca^2+ ^and Mg^2+^-free PBS (Invitrogen, Carlsbad, CA). Myocytes were then incubated with 0.1% Triton X-100 (in PBS) for 15 min at room temperature, which permeabilizes surface membranes as well as those of intracellular organelles (this step was omitted when fixing the cells only with methanol but was included in the two-step fixation protocol). Following washing (2 × 5 min) and blocking (5% goat serum in PBS; 2 × 10 min) the cells were incubated with primary antibodies (1 h at room temperature), washed (3 × 10 min in PBS-serum) and incubated with secondary antibodies (45 min at room temperature). Following 4 washes (with PBS; 10 min each) coverslips were mounted and viewed using confocal microscopy.

As a negative control, the primary antibody was adsorbed with the peptide against which it was made (when available). Negative staining controls (not shown) also included a null control, in which the primary antibody was omitted, which tested for non-specific staining of the secondary antibody. To avoid background from secondary antibodies alone, we normally pre-blocked the tissue with 5% normal serum from the same host species as the labeled secondary antibody. We used labeled secondary antibodies that have been pre-adsorbed against mouse and human and we titrated the labeled secondary antibody to obtain a maximal signal-to-noise ratio.

### Transfection of cells

The coding regions of Kir6.1 and Kir6.2 (a gift from Dr. S. Seino, Kobe University Graduate School of Medicine, Japan) were subcloned into pcDNA3. HEK-293 or COS-7L cells were cultured in D-MEM (Invitrogen) supplemented with 10% heat-inactivated fetal bovine serum and 20 μg/mL gentamycin. Cells were co-transfected according to the manufacturer's recommendations (Fugene 6, Roche Applied Science, Indianapolis, IN) with Kir6.1 or Kir6.2 cDNA (obtained from Dr S. Seino), SUR1 (Dr J. Bryan, Baylor College of Medicine, Texas) or SUR2A cDNAs (a gift from Dr Seino). Cells were lysed 48 h post-transfection.

### Preparation of mouse heart membrane fraction

Adult Sprague Dawley rats were anesthetized and the hearts were rapidly removed. Membranes were prepared essentially as described before [42]. The protein content was determined and equal amounts of proteins were subjected to Western blotting.

### Western blotting

Cells were solubilized in lysis buffer [25 mmol/L Tris, 150 mmol/L NaCl, 5 mmol/L EDTA, 1% (v/v) Triton X-100, 0.5% (w/v) deoxycholate, pH 7.5 supplemented with a cocktail of protease inhibitors (Sigma)]. After centrifugation (10 min at 16,000 g), an equal volume of 2 × Laemmli loading buffer was added to the lysate. Proteins were separated by 10% SDS-PAGE and transferred to Immun-blot PVDF membrane (Bio-Rad Laboratories, Hercules, CA). The membrane was blocked and incubated with primary antibody (see below). As secondary antibodies, we used HRP-conjugated anti-rabbit, anti-mouse IgG (Amersham Biosciences, Piscataway, NJ) or anti-goat IgG in TBS-Tween for 1 hour and detected using a chemiluminescent substrate (SuperSignal, Pierce Biotechnology, Rockford, IL).

### Antibodies

#### Antibodies against Kir6.1 subunits

Antibodies (NAF1) were raised against a peptide corresponding to residues 20–31 of the Kir6.1 N-terminus (ENLRKPRIRDRLP). There is a high degree of sequence similarity in Kir6.1 subunits between species in this region. We also raised a Kir6.1 antibody (CAF-1) to this peptide in chickens. In each case, a C-terminal cysteine was added for conjugation purposes. Peptides were synthesized and the antibodies were generated commercially (Quality Controlled Biochemicals, Hopkinton, MA). Each of these antibodies were peptide affinity purified. There is sequence similarity (81% identity) between this peptide and the recently-identified human beta-V spectrin. We tested for possible cross-reactivity of NAF-1 with beta-V spectrin using antibodies generously provided by Dr Jon Morrow (Yale University) and demonstrated that our antibodies had no cross-reactivity with beta-V spectrin (please see online Additional file 1). It should further be noted that the antibody epitope has no similarity with mouse beta-V spectrin (see UniGene Cluster Hs.198161).

Other anti-Kir6.1 antibodies that we attempted to use with varying degrees of success included the 78A antibody generated in the laboratory of Dr Tinker, which was raised in rabbits against a peptide corresponding to amino acids 399–420 of rat Kir6.1 with a terminal cysteine added for coupling purposes (RRNNSSLMVPKVQFMTPEGNQC) and the goat anti-Kir6.1 R-14 or C-16 C-terminal antibodies (sc-11224 and sc-11225; Santa Cruz Biotechnology, Santa Cruz, CA). In our hands, the 78A and R-14 antibodies failed to detect Kir6.1 protein in Western blotting of cardiac membrane fractions and did not appear to stain above background in immunohistochemistry assays (not shown).

#### Antibodies against Kir6.2 subunits

We used a goat anti-Kir6.2 G-16 antibody (sc-11228; Santa Cruz Biotechnology, Santa Cruz, CA) and an antibody (76A) developed in Dr Tinker's laboratory against a peptide (DALTLASSRGPLRKRSC) corresponding to a peptide within the Kir6.2 C-terminus (amino acids 357–372).

#### Antibodies against SUR subunits

We used a goat anti-SUR1 C-16 antibody (sc-5789; Santa Cruz Biotechnology) developed to an epitope mapping at the C-terminus and a goat anti-SUR2 C-15 C-terminal antibody (sc-5793; Santa Cruz Biotechnology). This antibody was initially sold as a pan-SUR2 antibody and was able to detect the SUR2A protein in Western blots of cell lysates from Kir6.2/SUR2A transfected cells, although with less sensitivity compared to SUR2B-transfected cells (data not shown). Currently, the antibody with the same catalog number is sold as being specific to SUR2B. We have not tested recent batches of this antibody for isoform specificity.

#### Other antibodies

Other antibodies used for immunolocalization included a mouse monoclonal α-actin smooth muscle antibody preconjugated to FITC (Clone 1A4; Sigma-Aldrich Corp, St. Louis, MO) and a rat monoclonal antibody (clone 3C4; BD Biosciences Pharmingen, San Diego, CA) raised against ICAM-2 (CD102), which is a cell surface glycoprotein constitutively expressed on vascular endothelial cells.

Secondary antibodies used included Cy3-conjugated donkey anti-rabbit IgG, Cy3-conjgated donkey anti-chicken IgY, Cy5-conjugated F(ab')_2 _fragment donkey anti-rat IgG and a Cy3- or Cy5-conjugated donkey anti-goat IgG (all from Jackson ImmunoResearch Laboratories Inc, West Grove, PA).

## List of abbreviations

Kir = Inward rectifying K^+ ^channel family

SUR = Sulphonylurea receptor

K_ATP _channel = ATP-sensitive K^+ ^channel

## Authors' contributions

AM, ER and JL carried out the immunohistochemistry experiments. PDC, SH and GL carried out the immunocytochemistry experiments. TN, AM, LP, HY, XT, JPG and AT participated in characterization of the antibodies. WAC, MA and TN participated in the design of the study. WAC conceived the study, participated in study design and coordination, and manuscript preparation. All authors made substantive intellectual contributions to the study, read and approved the final manuscript.

## Supplementary Material

Additional File 1 NAF-1 antibody does not cross-react with beta V spectrin. COS7L cells have been transfected with Kir6.1 cDNA and cell lystates were subjected to Western blotting with anti-beta V spectrin antibodies or NAF-1 anti-Kir6.1 antibodies. Click here for file
