# Supplementary figures and images for: Immunolocalization of KATP channel subunits in mouse and rat cardiac myocytes and the coronary vasculature
Source: BMC Physiol. 2005 Jan 12;5:1. doi: 10.1186/1472-6793-5-1 (PMC546210; doi:10.1186/1472-6793-5-1)

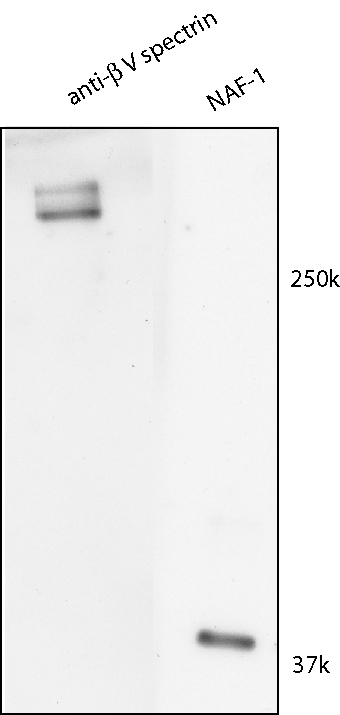

Supplement: Additional File 1 — NAF-1 antibody does not cross-react with beta V spectrin. COS7L cells have been transfected with Kir6.1 cDNA and cell lystates were subjected to Western blotting with anti-beta V spectrin antibodies or NAF-1 anti-Kir6.1 antibodies. [file 1472-6793-5-1-S1.jpeg]
